# Response to COVID-19: was Italy (un)prepared?

**DOI:** 10.1017/S1744133121000141

**Published:** 2021-03-05

**Authors:** Iris Bosa, Adriana Castelli, Michele Castelli, Oriana Ciani, Amelia Compagni, Matteo M. Galizzi, Matteo Garofano, Simone Ghislandi, Margherita Giannoni, Giorgia Marini, Milena Vainieri

**Affiliations:** 1 Business School, University of Edinburgh, Edinburgh, UK; 2 Centre for Health Economics, University of York, York, UK; 3 Population Health Science Institute, Newcastle University, Newcastle upon Tyne, UK; 4 SDA Bocconi School of Management, Bocconi University, Milano, Italy; 5 Department of Social and Political Sciences, Bocconi University, Milano, Italy; 6 Department of Psychological and Behavioural Science, LSE, London, UK; 7 Local Health Authority of Parma, Parma, Italy; 8 Department of Economics, University of Perugia, Perugia, Italy; 9 Department of Juridical and Economic Studies, La Sapienza University of Rome, Rome, Italy; 10 Department of Embeds, Management and Health Lab, Institute of Management, Sant'Anna Advanced School of Pisa, Pisa, Italy

**Keywords:** Health policy, COVID-19, National response, Italy

## Abstract

On 31st January 2020, the Italian cabinet declared a 6-month national emergency after the detection of the first two COVID-19 positive cases in Rome, two Chinese tourists travelling from Wuhan. Between then and the total lockdown introduced on 22nd March 2020 Italy was hit by an unprecedented crisis. In addition to being the first European country to be heavily swept by the COVID-19 pandemic, Italy was the first to introduce stringent lockdown measures. The SARS-CoV-2 outbreak and related COVID-19 pandemic have been the worst public health challenge endured in recent history by Italy. Two months since the beginning of the first wave, the estimated excess deaths in Lombardy, the hardest hit region in the country, reached a peak of more than 23,000 deaths. The extraordinary pressures exerted on the Italian Servizio Sanitario Nazionale (SSN) inevitably leads to questions about its preparedness and the appropriateness and effectiveness of responses implemented at both national and regional levels. The aim of the paper is to critically review the Italian response to the COVID-19 crisis spanning from the first early acute phases of the emergency (March–May 2020) to the relative stability of the epidemiological situation just before the second outbreak in October 2020.

## 1. Introduction

The SARS-CoV-2 outbreak and the related COVID-19 pandemic have been the worst public health challenge in recent Italian history, placing extraordinary pressures on the country's health care and long-term care systems, and on the economy as a whole.

Between 31st January 2020, when a state of national emergency was declared after the detection of the first two COVID-19 cases in Rome, and the easing of the stringent lockdown restrictions at the beginning of June, Italy was ‘hit by nothing short of a tsunami of unprecedented forces, punctuated by an incessant stream of deaths. […] Italy's biggest crisis since World War II’ (Pisano *et al*., 2020). Two months after the beginning of the first wave, the estimated excess deaths in Lombardy, the hardest hit region in the country, reached a peak of more than 23,000 deaths, equivalent to an excess mortality of +118% compared to the average mortality rate of the period 1 January–30 April 2015–2019 (Ghislandi *et al*., 2020; ISTAT, 2020).

In this paper, we aim to critically review the Italian response to the COVID-19 crisis spanning from the early acute phases of the emergency (March–May 2020) to the relative stability of the epidemiological situation just before the second outbreak in October 2020. In what follows, we first briefly describe how the Italian *Servizio Sanitario Nazionale* (SSN, National Health Services) is organised and the preparedness of the SSN before the epidemic started. Second, we describe the governance of the emergency set up by the government. Finally, we attempt a first assessment of the effects that the COVID-19 crisis had on the Italian health care system, separately addressing supply-side and demand-side considerations.

## 2. Overview of the Italian SSN

The Italian SSN was established in 1978 on the principles of universal access and free health care. The SSN is mostly financed through general taxation. In 2019, public health care expenditure was 74% of total health care expenditure (THE) and 6.5% of GDP. Private out-of-pocket payments account for 23% of THE, one of the highest in Europe, with the remainder coming from private health insurance. THE was 8.8% of GDP, in line with the Organisation for Economic Co-operation and Development (OECD) average, but almost 1% below the European Union (EU) (28) average and lower than most Western European countries; per capita spending was 3649 USD-PPP, 14% lower than the OECD average (Citoni *et al*., 2020).

Unlike other centrally managed national health care systems, the SSN has implemented a decentralised model since the early 1990s with complementary responsibilities allocated to the central and regional levels of government (Ferrè *et al*., 2014). The central government is responsible to set the overall funding requirements, goals and priorities of the SSN, mainly through the setting of ‘Essential Levels of Care’ (*Livelli Essenziali di Assistenza*, LEA) and by ensuring that the health expenditure of each region does not exceed the allocated budget. Nineteen regions and two autonomous provinces are responsible for the organisation and delivery of health care services – primary and secondary care, public health and social care – within their territories. They are allowed to do so with a high degree of administrative, political and also fiscal autonomy, e.g. regions can levy additional taxes to finance health care. This decentralised model has basically led to the development of 21 different regional/provincial health care sub-systems in Italy, with significant differences in terms of organisational and governance models (Nuti *et al*., 2016).

Over the last two decades the Italian SSN has experienced continuous financial cuts, which while guaranteeing financial sustainability, might have led to a number of negative and unintended effects. A cap to personnel costs in 2004, for example, led to a depletion of health care professionals' competencies (Noto *et al*., 2020). The 2007 bail-out plans in some regions resulted in the downsizing of hospital capacity, block of staff turnover and new co-payments on pharmaceutical expenditure (Bordignon and Turati, 2009; Piacenza and Turati, 2014). All of the above led to the realisation that more resources needed to be injected in the SSN, as recognised in the last national health plan ‘*Patto per la salute* 2019–2021’, issued in December 2019, just before the COVID-19 crisis (Ministero della Salute, 2019).

## 3. National plan for preparation and response to an influenza pandemic

By the impact observed in the early stage of the emergency, it is plausible to believe that the pandemic caught the population, the local and national governments and even the international organisations and public health experts largely unprepared. However, since the H5N1 avian influenza re-emergence in the Far East in 2003, the risk of a flu pandemic in the country was seen as concrete and persistent. In 2005, the WHO issued a list of recommendations (WHO, 2020) for updating and developing a national pandemic plan, following which the Ministry of Health in Italy developed a ‘National Plan for Preparation and Response to an Influenza Pandemic’ (Ministero della Salute, 2006), defining objectives and activities, agreed with the regions, to be carried out to prevent and cope with a future pandemic throughout the national territory. The general objective of the plan was to strengthen preparedness for an epidemiological emergency at the national and local levels, and more specifically to (i) quickly identify, confirm and describe cases of influenza caused by new viral subtypes, in order to promptly recognise the onset of the pandemic; (ii) minimise the risk of transmission and limit morbidity and mortality due to the pandemic; (iii) reduce the impact of the pandemic on health and social services and ensure the maintenance of essential services; (iv) ensure adequate training of personnel involved in the response to the pandemic; (v) ensure up-to-date and timely information for decision makers, health professionals, the media and the public and (vi) monitor the efficiency of the interventions undertaken. However, the plan was never revised in the 14 years since its development. This attracted strong criticisms, as reported also in the media (Puente, 2020).

A number of key intermediate steps were envisaged to achieve these objectives, which, as unfortunately reported during the beginning of the COVID-19 pandemic (de Maria; Palladino, 2020), remained largely unfulfilled. Even the Italian Prime Minister, on 24th February, threatened to take back powers from the regions and autonomous provinces because they were ‘in charge of implementing healthcare but not prepared to face a national emergency’ and complained about the lack of application of ‘unspecified’ preparedness protocols (Carinci, 2020). This gap does not come unexpected when considering that since 2001 in the allocation of the national health care budget no more than 5% was earmarked for prevention, which also includes the pandemic preparedness activities, with community and hospital care (51 and 44%, respectively in 2019) gaining the lion share (CERGAS Bocconi, 2019).

More recently the Ministry of Health developed a new National Preparedness Plan (PanFlu 2021–2023), which identifies all the necessary actions that ought to be followed to avoid the health care system being overwhelmed again (Ministero della Salute, 2021). It also includes the possible responses to be taken in the event of a future epidemic, such as appropriate chain of commands, prevention and control measures. Furthermore, the new plan includes an explicit monitoring function of the implementation of the plan itself (Fassari, 2021).

## 4. Governing the emergency: who? when? how? why?

On 31st January 2020, the Italian Government declared a national emergency in order to be able to face the incoming COVID-19 crisis. The most direct consequence of this act was that the Department of Civil Protection, an operative branch of the Presidency of the Council of Ministers, took up the important role of coordination and execution of the emergency intervention. The Civil Protection is a highly regarded and reputable organisation, a reputation matured through their long experience with dealing with earthquakes and other national disasters.

Additional committees and roles were also created to front the emergency. In particular and following the timeline of events, on 3rd February, the Civil Protection Department set up a technical and scientific committee (hereafter CTS), comprising of high level civil servants from within the Ministry of Health, the *Istituto Superiore di Sanità* (National Institute of Health, ISS), regional governments and the same Civil Protection Department, as well as clinical experts (public health experts, virologists and clinicians) to provide scientific advice to the government. The regional governments of Lombardy, Veneto, Emilia-Romagna and Piedmont, all at the epicentre of the COVID-19 health crisis in Italy, also set up their own task forces and advisory committees. Furthermore, on 17th March, to respond to the inadequate availability of both personal protection equipment (PPE) and ventilators, the Prime Minister appointed a commissioner in charge of coordinating their centralised procurement. Finally, on 10th April, a ‘committee of experts in economic and social subjects’ was established by the Prime Minister to develop plans and guidelines for the transition from total lockdown to the wider reopening of the country.

The state of emergency had two important implications for the governance of the crisis. First, to guarantee a quick response, the government was allowed to bypass the Parliament in the definition of legislative interventions. The government did so by approving so-called ‘decrees of the Prime Minister’. This approach, although legally grounded in the Italian law, blurred the boundaries between the executive and the legislative powers, *de facto* freezing the Constitutional framework. For this reason, critics have questioned the decision by the government to prolong the state of emergency first until the 31st October and then until the 31st January 2021. Second, the state of emergency introduced the possibility of derogation of existing procurement rules. Italy has very strict procurement rules and the national anti-corruption agency is dedicated to check the legitimacy of procurement bids. The Department of Civil Protection issued new procurement regulations to be valid mainly for the acquisition of PPE, tests and ventilators, simplifying and accelerating the existing procedures.

## 5. National and regional policy responses

The first Italian COVID-19 positive patient was reported by the local health authorities in Lombardy in Codogno (Lodi) on 20th February, followed by a number of additional cases in the neighbouring areas of Emilia-Romagna and Veneto. On 21st February, the first COVID-19 death was recorded in Veneto. On 23rd February, the government reacted by introducing the first movement and access/exit restrictions around these hotspots, known as ‘red zones’. Additional restrictive measures for the whole of Lombardy, Veneto, Emilia-Romagna, Friuli-Venezia Giulia, Liguria and Piedmont followed on 25th February. Nationwide closure of schools and universities was declared on 4th March, with additional social distancing measures introduced on 9th March. A national partial lockdown was enforced on 11th March, affecting bars, restaurants and recreational facilities, and culminating in the complete lockdown on 22nd March. Further restrictions to people's movements were introduced on 25th March, except for essential reasons (e.g. work, health and getting supplies).

Phase 1 of the Italian response to the emergency ended on 3rd May. This was followed by phase 2 (4th May to 2nd June) during which most primary and secondary productive sectors, professionals and private health care clinics and most retail shops, businesses and customer services, resumed activities subject to sector-specific COVID-19 safety protocols (DPCM, 2020b). Previous restrictions to the free movement of citizens were lifted, within one's region of residence only. Further regulations, relaxing the existing lockdown measures, were adopted nationwide and locally on 17th May (DPCM, 2020a). At this stage, a key role was played by the Italian regions, which asked and obtained the right to set specific regional guidelines (de Belvis *et al*., 2020).

As of 3rd June, all businesses reopened subject to aforementioned protocols and social distancing rules. Free inter-regional movement of citizens was also reinstated, albeit with restrictions on foreign travel. The wearing of face-masks (for ⩾6 years) outdoors, on public transport, in shop/businesses became mandatory as well as keeping at least 1 m distance. Schools and universities remained closed, but provided lessons through distance learning platforms (*didattica a distanza*) and reopened only in September 2020 in a staggered way.

The outbreak of SARS-CoV-2 has unevenly affected Italian regions, with a clear north-south gradient. These differences are mainly due to multiple independent entries of the virus in northern Italy, but may also be linked to the diverse set of policies implemented at the regional level. In a companion paper, we discuss the differences and similarities of the Italian regional policy responses to COVID-19.

Similarly to many European countries and the USA, nursing homes (*Residenza Sanitaria Assistenziale*, RSA) in Italy were particularly hit by the COVID-19 outburst (Berloto *et al*., 2020; Ravizza, 2020). The excess mortality recorded in these settings seems to have followed the regional pattern of exposure to and incidence of the SARS-CoV-2 outbreak. These excess deaths, flagged up by a newspaper investigation, prompted the ISS to conduct a targeted survey, which revealed that an average of 9.1% of all nursing homes’ residents died in Italy, with a peak of 14% in Lombardy, between 1st February and 14th April, of which about 37.4% officially due to COVID-19 (Istituto Superiore di Sanità, 2020).

A timeline with summary of the Italian national policy responses until the beginning of the second outbreak in October is reported in Table 1.

**Table 1.** Timeline of main COVID-19 events and responses undertaken by the Italian government

Date	Main events and policy responses
31-01-2020	The Italian Council of Ministers declares a 6-month national emergency handing the coordination of the COVID-19 emergency responses to the Head the Civil Protection Department, following the detection of the first two COVID-19 positive people in Rome – two Chinese tourists travelling from Wuhan
21-02-2020	Detection of the first Italian COVID-19 positive patient in Lombardy, followed by further cases in neighbouring regions of Emilia Romagna and Veneto
23-02-2020	The Government establishes two ‘lockdown (red) zones’ in both Lombardy and Veneto, with the imposition of the first movement restrictions – travelling from home must be justified on either health, work or ‘necessity’ grounds, e.g. grocery shopping
25-02-2020	Further restrictive measures introduced in Emilia Romagna, Friuli Venezia Giulia, Liguria, Lombardy, Piedmont and Veneto, incl. school closures in Friuli Venezia Giulia, Lombardy and Veneto; whilst in other regions mentioned and Calabria, schools closed only for 1 week
27-02-2020	A National Surveillance system, coordinated by the ISS was set up to oversee the collection and collation of daily data from regions and from ISS’ National Laboratory for SARS-CoV-2 through a web portal
01-03-2020	Creation of ‘red zones’: 10 municipalities in Lombardy, incl. Codogno (first town to be put in lockdown); Veneto, incl. Vo’ (first town to be put in lockdown); other provinces in Emilia Romagna, Marche and Liguria
04-03-2020	Closure of schools (all stages) and universities in the whole country
08-03-2020	Extension of lockdown restrictions to all Lombardy and large areas of Northern Italy
09-03-2020	Partial lockdown restrictions extended to the whole of Italy
11-03-2020	Closure of restaurants/bars/pubs, except for essential services
17-03-2020	Decree (no. 18/20, ‘Cura Italia’), which included measure to bolster the SSN, support employment and suspend fiscal obligations
22-03-2020	Interruption of all non-essential productive activities: complete lockdown
25-03-2020	Further restrictions imposed to people's movements
27-03-2020	Peak in the reported number of daily deaths (969)
08-04-2020	Decree (n. 23/20, ‘Liquidità’), which included temporary measures to facilitate access to loans, support business continuity and corporate liquidity and measures to support export, internationalisation and business investment
04-05-2020	Reopening of most factories and various wholesale businesses, within pre-set health safety protocols; possibility to visit close relatives
13-05-2020	Decree (n. 34/20, ‘Rilancio’), which included among others measures to support and bolster hospitals and community-based health care services, increase the number of nurses and reinforce medical education; support business activities and further postpone fiscal obligations; support employment; extend school closure
18-05-2020	Reopening of bar, restaurants, shops and some social activities (e.g. public worship)
04-06-2020	Resuming of unrestricted inter-regional movement
15-06-2020	Resuming of recreational activities (e.g. cinemas and holiday camps)
29-07-2020	Extension of the state of emergency to 31st October
07-10-2020	Extension of the state of emergency to 31st January 2021
24-10-2020 (addressing the second wave)	New restrictive measures to people's movement (public squares to close at 9 pm, curfew from 11 pm to 5 am in some regions); closures of restaurants/bars/pubs from 6 pm; online schooling for 75% of secondary schools and university students; closures of cinemas/theatres/music halls; only outdoor sports/activity is allowed, except for elite sports

## 6. Supply and demand side considerations of the COVID-19 response in Italy

Addressing the emergency required interventions on both the supply and demand of health care services, in this section, we attempt a first assessment of the effects that the COVID-19 crisis had on the Italian health care system, separately addressing supply-side and demand-side considerations.

### 6.1 Supply-side considerations

The outbreak of a health crisis, especially at a scale like the COVID-19 crisis, puts even the best health care systems under increased pressures and stress. In this section, we provide a summary of the various measures introduced by the Italian central government to increase physical infrastructure capacity and address the emerging workforce shortages. However, the COVID-19 crisis has also been instrumental in Italy, as in many other European countries, in fostering the adoption of already existing digital technology by shifting to, and increasing the offer of, telemedicine, for example.

#### 6.1.1 Physical infrastructure and devices

Increasing production capacity is normally difficult in the short term, but what has been achieved under the urgency of the COVID-19 crisis has been unprecedented for Italy. The expansion in the supply side has entailed the rapid conversion or building of new hospital facilities and intensive care units (ICU) for the care of COVID-19 patients, the procurement of massive quantities of PPEs and other medical devices, and the hiring of additional health care workers.

#### 6.1.2 Hospital facilities and ICU beds

One of the key bottlenecks of the recent COVID-19 crisis has been the lack of adequate ICU facilities. The total number of ICU beds available in the SSN was increased by almost 65% during the acute phase of the response, equivalent to about 3360 additional beds, from around 5300 in 2018 (Aimone Gigio *et al*., 2020). A further 30% expansion (almost 2400 beds) to the already expanded ICU bed numbers has been planned (April 2020) which, if completed, will result in a more than doubled overall pre-pandemic capacity. The growth in the number of ICU beds applied to all regions, but not homogenously, as their needs were dependent on the epidemiological development and severity of the COVID-19 outbreak in each region. The most recent increases in the number and locations of COVID-19 infections have highlighted how the virus has travelled across the whole length of the Italian peninsula, facilitated by the easing of the restrictions on movement and the summer holiday season. This has brought to the fore that, despite the recent efforts in increasing ICU capacity, this bottleneck still remains a critical issue in areas previously not affected.

#### 6.1.3 PPEs and medical devices

PPE, tests, contact tracing and other medical devices have been essential for enabling an effective response and an efficient prevention of the spread. Since the start of the outbreak, the Civil Protection Department has been coordinating the procurement and distribution of their supplies to the regions. Despite an initial dramatic shortage in PPEs and other devices, 52 contracts with national and international sellers were activated, for a total amount of about 357 million EUR, to buy 350+ million masks, 7.2+ million gloves, 107,000+ protective suits, 100,000+ protective glasses, 2500+ mechanical ventilators and 400 oxygen flow-meters (Dipartimento della Protezione Civile, 2020). Tenders were launched and adjudicated in record time thanks to all stakeholders involved working around the clock. In addition to national supplies, regions, local administrations and hospitals have also proceeded to direct purchases or procurement of these goods through other channels (e.g. donations).

#### 6.1.4 Workforce shortages

The effort to expand production capacity to be able to treat COVID-19 patients has been accompanied by the need to stop most elective health care activities and shift personnel to the acute and intensive care of COVID-19 patients. This has meant also increasing working hours and length of shifts for medical and nursing staff on duty. The emergency magnified, therefore, all the negative consequences of the extensive underfunding of the Italian health care system and of the stop in workforce turnover. Italy, as many other OECD countries, had been suffering from both workforce shortages in the health care sector (CERGAS Bocconi, 2019) and an ageing medical workforce (OECD, 2019) for some time. According to the OECD Health at a Glance Indicators (OECD, 2019), in 2017 Italy had the largest share (55 vs 34% OECD average) of medical doctors aged 55 and over among all OECD countries – a share which increased by 36% between 2000 and 2017. However, the issue of workforce shortages is especially true in the case of nurses. Italy has in fact fewer nurses than nearly all western European countries (excluding Spain), with 5.8 nurses per 1000 population compared to the European average of 8.5. And while Italy fares better compared to their European peers in terms of medical professionals (4.0 doctors per 1000 population compared with the EU average of 3.6), Italy has the lowest doctor-to-nurse ratio in the OECD (1.4 nurses per doctor) (OECD/European Observatory on Health Systems and Policies, 2019). Despite recent attempts by the Italian government to address this imbalance through increasing the number of students training to become nurse (the number increased to 13,000 in 2014 from a low 3100), the COVID-19 crisis has heightened the shortage of health care professionals suffered by the SSN, with a pre-COVID-19 incidence of medical personnel of about 95 workers per 10,000 inhabitants (57 nurses, 19 doctors and 19 other technical staff).

The shortage of nurses and medical doctors front lining the emergency response, also in light of the high number of nurses and medical professionals testing positive to COVID-19 (Bellizzi *et al*., 2020), forced the Government to introduce several measures (DL, 2020a, 2020b; DPCM, 2020b) in order to face the rapidly rising demand of extra medical and other health care personnel. These measures included inter-regional redistribution of health care personnel, the re-hiring of retired medics, nurses and other health care professionals, the creation of faster recruitment tracks, the possibility to employ personnel on a freelance basis, the hiring of 20,000 health care professionals (3.5% growth in the health workforce): the new hires comprised of more than 4300 additional medical doctors, mainly anaesthesiologists; around 9700 nurses; and 6000 other health care professionals, mainly technical personnel (Aimone Gigio *et al*., 2020).

Further measures included the allocation of 250 million EUR to pay for staff overtime, the possibility for health care facilities to postpone retirement for eligible staff, the possibility for retired doctors and nurses to return to practice on a voluntary basis (in the peak of the crisis in Lombardy, more than 300 retired doctors and 500 retired nurses returned to practice on a voluntary basis), and to request the temporary enrolment of doctors and nurses from the armed forces. Furthermore, hospitals were given the possibility to recruit on a freelance basis, doctors and nurses not yet listed in the Medical Register and medical doctors and nurses practicing abroad under EU directives have also been allowed to work in Italy on a temporary basis.

#### 6.1.5 The shift to digital care

During the COVID-19 health crisis, as people were asked to shelter-in-place, health care systems had to quickly move to other, innovative, forms of providing continued care to the population, especially the elderly and those affected by chronic conditions. This lead to a forced acceleration in the adoption of telemedicine, e-prescribing and similar practices. In Italy, especially for community care services, many regions activated a number of alternative provisions of health care, such as teleconsultations, over a very short period of time. Telemedicine in Italy has been traditionally delivered using several applications, poorly interconnected and with inconsistent local and regional reimbursement practices. A temporary model to ease organisational aspects of the implementation of telemedicine services during the emergency was issued by the ISS, albeit without a specific national guidance on reimbursement codes, which caused the occurrence of inter-regional differences. At the start of the pandemic, telemedicine was not explicitly covered in the guaranteed LEA. Although private telemonitoring service providers reported a marked increase in the use of direct-to-consumer services, the lack of a framework to reimburse telemedicine services hindered wider-scale adoption by many public institutions (Petracca *et al*., 2020).

The COVID-19 crisis boosted the digitisation process in Italy, thanks to a relaxation of usual red tapes (General Data Protection Regulation (GDPR), procurement rules and organisational resistance) in two ways. First, it helped revitalise dormant innovations, like e-prescription and telemedicine already in place (Oliveira Hashiguchi, 2020), given the urgency of offering alternative ways to care for patients. This forced many previously reluctant or late ‘bloomer’ professionals to swiftly rely on telemedicine. Second, it led to further investments in technological infrastructure necessary to tackle the challenges posed by the outbreak, such as the introduction of mobile apps for controlling contact tracing or social distancing (Kummitha, 2020). The *Immuni* app was introduced as a surveillance system, albeit not compulsory, in June 2020. Two months later, only 4 million Italians (about 6% of the total population) had downloaded it, against the minimum required threshold of 12 million (equivalent to 20% of total population downloads or 60% of actual users) to guarantee its real effectiveness (Berra, 2020; Ferretti *et al*., 2020). The most recent estimates report over 10 million downloads of the Immuni app since its launch (Immuni Italia, 2020).

### 6.2 Demand-side considerations

The surge of COVID-19 patients who required medical care, and specifically intensive care, has led almost everywhere to a rapid and extensive reprogramming of health care service delivery (Remuzzi and Remuzzi, 2020). Most regional health care systems have seen the capacity to treat surgical patients decrease dramatically because of the reallocation of resources to the pandemic response, and following government's advice to suspend all non-urgent elective surgery (Mayol and Fernández Pérez, 2020). In Italy, it has been estimated that a weekly total of 50,552 operations have been cancelled over the 12-week period of peak disruption. Figures about cancellation rates have been placed at over 90% for benign surgeries (e.g. hip and knee replacements), and between 20 and 29% for obstetrics and cancer surgery (CovidSurg Collaborative, 2020). Similarly, outpatient procedures have been put on hold to free up staff and resources to cope with COVID-19 patients. People with chronic diseases faced changes in their usual standards of care and protocols (Kohli and Virani, 2020; Schrag *et al*., 2020).

A report by *Nomisma*, an Italian business consultancy company, estimated that during the COVID-19 emergency, 75% of elective surgeries was postponed. Out of 410,000 surgeries that needed to be rescheduled, 135,700 (33% of the rescheduled surgeries and 79% of the specific DRG) refer to surgeries of the muscular system and connective tissue; 54,000 (13% of the rescheduled surgeries and 56% of the specific DRG) refers to surgeries of the cardiovascular system and 39,800 (10% of the rescheduled surgeries and 65% of the specific DRG) refers to surgeries of the digestive system. The remaining 180,000 surgeries refer to the rest of the major diagnostic groups, heavily affected by the emergency with between 70 and 97% rescheduled surgeries (Nomisma, 2020; quotidianosanità.it, 2020).

Finally, weeks of nationwide lockdown had also an effect on the number of people presenting to A&E and subsequent emergency admissions. A retrospective analysis of patients admitted for acute coronary syndrome at 15 hospitals in northern Italy during the early days of the COVID-19 outbreak revealed a mean rate of 13.3 emergency admissions per day compared to the 18.9 admissions during the previous year (incidence rate ratio, 0.70; 95% CI, 0.63 to 0.78; p < 0.001) (De Filippo *et al*., 2020).

## 7. Discussion

Was Italy unprepared? Answering this question is neither simple nor straightforward.

Italy was the first EU country to be hit by the COVID-19 epidemic. The initial response from both the Italian national and regional governments, business organisations, as well as that the general public was one of disbelief and inaction. However, the declaration of an emergency state early on in the crisis enabled the national government to take immediate and executive decisions to tackle the health crisis. In this midst, the decision to impose strict lockdown measures, similar to those imposed by China, was a difficult exercise as it required dealing with the unprecedented trade-off between enforcing measures that impinge on individual liberties in a democratic system, and the need to contain, or at least mitigate, the spread of the virus. Delays in the enforcement of lockdown measures, especially in closing non-essential production activities, ensued because of the need to acquire the consensus of both business industry and union representatives (Bosa *et al*., 2020).

Moreover, the early phase of the emergency was characterised by low level of compliance with and adherence to public health measures. An example of it is the mass flow of people who travelled from the hardest hit northern regions towards the south, before the introduction of the national lockdown in March, after this policy was prematurely leaked to the press, and which may potentially have had a negative impact on the spread of the outbreak in previously unaffected areas (Bosa *et al*., 2020). However, most southern regions took immediate steps to deal with the flux of people coming from the northern regions, by for example introducing a 14 day self-isolation period for people travelling from the hardest hit regions. This may have allowed these regions to keep their initial numbers of positive COVID-19 cases low(er) and to ‘flatten the curve’ earlier than northern regions (Bosa *et al*., 2020). The second wave of the pandemic is not sparing southern regions, revealing pre-existing weaknesses in their models of health care organisation and delivery.

Italy's multi-level governance structure and its decentralised health care system have allowed local governments (both regional and municipalities) to tailor their responses to the needs of their population and to proactively adopt further measures as required by the epidemiological development of the COVID-19 outbreak in their respective areas. However, we think that ‘this pluralism might have impeded faster and more integrated responses, and may have fuelled inter-governmental tensions’ (Bosa *et al*., 2020).

The lack of a brushed up emergency plan, the mismanagement of ‘patient 1’ and other initial cases, insufficient transparency in communication and a surveillance system initially ill-suited to allow for the central coordination of the national emergency, all conspired to make the first response to the COVID-19 crisis mainly a hospital-centred response, especially in Lombardy. This proved to be suboptimal, as revealed by the high incidence and high rate at which the virus spread, and the subsequent high need for ventilators and intensive care beds. Italy appeared to be under-equipped with respect to both, which has been suggested to have put the country under great(er) risk during this crisis. A study by Remuzzi and Remuzzi (2020), for example, estimated that by 29th March between 9 and 11% of COVID-19 patients required ICU care. Given the number of COVID-19 cases was at the time equal to 73,800, the authors stated that around 7380 ICU beds were needed, a number well exceeding the ICU bed capacity at the time. This may have forced ICU specialists to prioritise patients more likely to benefit and survive.

Furthermore, in the urgency of addressing COVID-19 patients in a hospital setting (with the doubling of ICU beds in the span of a mere 15 days), the national and regional governments might have been slow in organising an equally effective response at the primary/community care level. The high level of infected general practitioners (as compared to all health care professionals) testifies the lower level of attention that this part of the health care system received in the overall COVID-19 emergency response strategy (FNOMCeO, 2020).

However early on in the pandemic (March 2020), the central government required the creation of special units (*Unità Speciali di Continuità Assistenziale*, USCA, 1 every 50,000 inhabitants) to manage COVID-19 patients, who did not require hospital care, to be followed in the community, including the monitoring of people in home-isolation (DL, 2020a, 2020b). However, their introduction by the single regions has not been uniform with a minimum of five USCAs activated in Molise and PA Bolzen against the maximum number of 250 USCAs in Lazio. Lombardy, the region that has historically invested the least on primary/community care, has only activated 157 USCAs against the scheduled 202 (Fassari, 2020).

Another important issue – raising questions on the appropriateness and the timing of the containment measures implemented in Italy, and especially in the Lombardy region – is in the number of deaths in nursing homes. In Lombardy, for example, to ease pressure on hospitals already working at full capacity, regional decision makers proposed to use RSA beds to treat non-critical COVID-19 patients, provided that these patients could be properly isolated (Lombardy Region, 2020). At the time of writing, these decisions are under judicial investigations to establish whether they have contributed to the excess deaths observed in nursing homes in Lombardy (Serra, 2020). Furthermore, RSA's staff and carers have suffered from massive shortage of PPE, similarly to other countries (Berloto *et al*., 2020), and from a widespread lack of appropriate training. Finally, the COVID-19 crisis has highlighted the need to review the way social care is organised and delivered in Italy, and the need for a regulatory body responsible for controlling the quality of services provided by RSAs, which does not currently exist (Ravizza, 2020).

Disruption or deliberate delays in seeking needed care may have raised concerns, anxiety, fear and may have negatively impacted the mental health of patients, especially those in vulnerable and fragile groups (Citoni *et al*., 2020). A silent sub-epidemic of people who needed hospital care but did not dare to show up may well rival the carnage directly produced by COVID-19 – a collateral damage from delay of less urgent care that truly could not wait, with potentially thousands of missed diagnoses who will deteriorate or appear as late presentations or inoperable. The toll of unaddressed health problems is accompanied by a mounting backlog of procedures that could cost billions to the SSN and may require a substantial amount of extra health care workforce input to bring it under control (Rosenbaum, 2020).

Finally, it still needs to be investigated what the effects on access to care and the quality of care provided by the move to digital health care provision are, given that not all types of patients nor all types of clinical conditions are amenable to be treated as such.

Despite this unprecedented injection of digitalisation, there are still professionals and patients who still have to catch-up – an issue termed ‘The Great Digital divide’ – making it a priority to bring people who are ‘digitally excluded online’ (Capgemini Research Institute, 2020).

## 8. Conclusions

The first wave of the COVID-19 outbreak and related deaths were mainly concentrated in the northern regions, the second wave has spread more widely geographically, facilitated by the easing of the lockdown restrictions, especially on movement, implemented in June and subsequent summer holidays. Current government instructions are based on a three-tier regional risk assessment. At the time of writing, Italy was still experiencing a daily rise of COVID-19 cases and related deaths, which led to the re-introduction of movement restrictions, curfews and early daily closures of businesses. When and how these restrictions will impact curbing and flattening the curve is a question that cannot be answered yet.

The COVID-19 pandemic has hit the country after years of strict spending reviews and severe cost containment measures (at least since the 2008 economic crisis). These have cut down resources to the health system and hospital capacity, with said cost-containment measures shifting *de facto* the burden of health care finance from national and regional governments to households (e.g. increasing user charges), and setting tighter budget constraints for pharmaceutical public expenditure, in a context of increasing socioeconomic inequalities in both health care use and financing (Citoni *et al*., 2020).

In 2019, the Italian government started to redress the financing of the SSN, increasing its funding to 114.5 billion EUR (+1.59% increase in nominal terms), with further forecasted increases of 2 billion EUR and 1.5 billion EUR for 2020 and 2021, respectively (Citoni *et al*., 2020; de Belvis *et al*., 2020). Moreover, since the outbreak of the COVID-19 pandemic and the subsequent economic crisis, the government approved a series of economic measures aimed at supporting several sectors of the Italian economy, including health care (de Belvis *et al*., 2020).

The main upcoming challenges, besides adequate and sustainable funding of the SSN, for the Ministry of Health, the national government and the regions are how to reorganise the SSN, possibly even in its governance; what priorities to set to provide and strengthen health care services (e.g. prevention/public health, primary/community care); how to overcome workforce shortages in the medium and long term, assuring the right mix of competences within the SSN; and how to modernise the physical infrastructures of the health care system; all the while keeping in mind, that reinforcing the country's preparedness to future epidemics can no longer be postponed.
